# Mast cell activation and clinical outcome in pediatric cholelithiasis and biliary dyskinesia

**DOI:** 10.1186/1756-0500-4-322

**Published:** 2011-09-06

**Authors:** Craig A Friesen, Nancy Neilan, James F Daniel, Kim Radford, Jennifer V Schurman, Ding-You Li, Linda Andre, Shawn D St Peter, George W Holcomb

**Affiliations:** 1 Department of Pediatrics, The Children's Mercy Hospital, 2401 Gillham Rd., Kansas City, Missouri, USA; 2 Department of Surgery, The Children's Mercy Hospital, 2401 Gillham Rd., Kansas City, Missouri, USA

**Keywords:** mast cell, biliary dyskinesia, cholelithiasis, cholecystectomy

## Abstract

**Background:**

The current study was undertaken to determine the degree of activation of gallbladder mucosal mast cells, whether mast cell (MC) density or activation differ between patients with and without a positive clinical response to cholecystectomy, and whether either density or activation correlate with gallbladder emptying.

**Results:**

Fifteen biliary dyskinesia (BD) and 13 symptomatic cholelithiasis (CL) patients undergoing cholecystectomy were prospectively enrolled. Gallbladder wall MC density (by immunohistochemistry) and activation (by electron microscopy) were determined. Clinical response was evaluated 30 days post-cholecystectomy on a 5-point Likert-type scale. A complete or nearly complete clinical response was seen in 100% of CL and in 87% of BD patients. The overall degranulation indices were 49.4 ± 18.7% for CL patients and 44.2 ± 16.8% for BD patients. Neither MC density nor activation correlated with the gallbladder ejection fraction. A complete clinical response was associated with lower epithelial MC density.

**Conclusion:**

Cholecystectomy is efficacious in relieving pain in both CL and BD patients. BD and CL are associated not only with increased MC density but a moderate to high degree of MC activation. A possible relationship between MC density and outcome for BD warrants further investigation.

## Background

The Rome III working group defined a number of functional gastrointestinal disorders in adults and children [1,2]. Functional gallbladder disorder (FGD), previously known as biliary dysfunction) was included in the adult but not the pediatric diagnoses. FGD is defined by recurrent symptoms of pain in the epigastrium or right upper quadrant lasting 30 minutes or longer and occurring at different intervals, among other criteria [3]. Support for this diagnosis is provided by an abnormal gallbladder ejection fraction with a normal hepatobiliary ultrasound and laboratories [3]. For children, the entity which would most closely resemble FGD has been termed biliary dyskinesia. Biliary dyskinesia (BD) and cholelithiasis (CL) are the most common indications for cholecystectomy in children and adolescents [4-6].

Although there has been extensive evaluation of the pathophysiology of cholelithiasis, the pathophysiology of FGD or BD has not been well described. Previously we evaluated gallbladder wall inflammatory cells in children with symptomatic CL and BD [7]. These two conditions were associated with a 9 to 12-fold increase in mucosal MC density. This parallels other functional gastrointestinal disorders, specifically functional dyspepsia and irritable bowel syndrome, which have been associated with increased MCs in the stomach, small bowel, and/or large bowel [8-11].

While we have previously demonstrated increased MC density in BD and symptomatic CL, this is not sufficient alone to implicate MCs in the pathogenesis or generation of symptoms in these patients. A large part of the biologic activity of MCs is attributable to released mediators which are active in a concentration-dependent fashion [12]. Thus, the biologic activity of MCs is defined not only by density but by the degree and location of activation. This distinction can be demonstrated by previous reports implicating MCs in the pathogenesis of IBS in adults. IBS has been associated with increased MCs in the terminal ileum, cecum, proximal descending colon, and rectum [9-11,13]. IBS patients also demonstrate increased density of activated or degranulating MCs as compared to controls and in IBS patients, MCs in close proximity to nerves are more likely to be activated [11,13]. Only the MC density in close proximity to nerves is correlated with the severity and frequency of pain in these patients [11].

If MCs are to be implicated in the pathogenesis of BD or symptomatic CL, not only do MCs need to be increased in density but it needs to be demonstrated that MCs are actively releasing mediators. The current study was undertaken to assess the degree of gallbladder mucosal MC activation. In addition, this study evaluated whether MC density or activation differ between patients with and without a positive clinical response to cholecystectomy.

## Methods

This was a prospective study evaluating gallbladder histology (specifically MC density and activation) and post-operative clinical response in patients undergoing laparoscopic cholecystectomy between February, 2006 and September, 2008 for symptomatic CL and BD. The study was approved by the Institutional Review Board at Children's Mercy Hospital in Kansas City and written consent/assent was obtained for all participants prior to participation in the study.

### Patient Selection

Patients were eligible for enrollment if they had right upper quadrant (RUQ) or epigastric pain of at least 8 weeks duration from the onset of the first bout and were scheduled for cholecystectomy. BD patients were required to demonstrate delayed gallbladder emptying (EF < 35%) following CCK stimulation on a single cholescintigraphy. Reproduction of pain during cholescintigraphy was not considered. CL patients were required to have the presence of a gallstone within the gallbladder on ultrasound.

Patients were not excluded for pain in other regions in addition to the RUQ or epigastric pain. Patients were excluded from either group for evidence of biliary obstruction defined as direct bilirubin ≥ 1.0 mg/dL on any previous laboratory evaluations or common bile duct diameter ≥ 7 mm on any previous ultrasound. Patients were excluded if they had received anti-inflammatory medications or had a history of inflammatory bowel disease, rheumatologic disease, or cancer. In addition, BD patients were required to exhibit normal filling (< 1 hour)on cholescintigraphy.

### Histology

Sections from the body of the resected gallbladders were embedded and processed in the usual fashion including staining with hematoxylin and eosin for the usual evaluation by a board certified pediatric pathologist. Additional specimens were obtained for immunohistochemical (IHC) staining for MCs. For IHC evaluation, serial 3- μm paraffin sections were air dried and fixed on slides. The sections were deparraffinized using a xylene substitute and then rehydrated in alcohol to tris-buffered saline. Endogenous peroxidase activity was blocked using 3% hydrogen peroxide followed by a protein block with 5% goat serum. Residual biotin and avidin activity were quenched using avidin and biotin block, respectively. Monoclonal mouse anti-human mast cell tryptase, Clone AA1 was used as the primary antibody and applied as a 1:1000 dilution for 1 hour at room temperature (18°C - 25°C). Labeled streptavidin-biotin (LSAB) was used for the detection system with diaminobenzidine tetrahydrochloride (DAB) as the chromogen. Sections were counterstained with hematoxylin. MCs were enumerated for 10 high power fields per layer (epithelium, lamina propria, and muscularis mucosae) by a single investigator (NN). The largest MC count in any 40X high power field (hpf) was reported as the peak and the average number/hpf was reported as the mean.

### Electron Microscopy

Specimens for electron microscopic (EM) evaluation were obtained in the operating room immediately following cholecystectomy and were placed in 2% glutaraldehyde in 0.1 M sodium cacodylate, pH 7.4. All specimens were taken from the body of the gallbladder and were approximately 5 mm × 5 mm. Specimens were post fixed in osmium tetroxide. The tissue was then dehydrated in graded alcohols and embedded in epoxy resin. Sections were cut at 80 nm thickness and contrasted with uranyl citrate and lead citrate. Transmitting EM was performed and MCs were identified and photographed at 4800X. The photographs were then evaluated and the MC degranulation index was calculated by previously described methodology [14]. The number of granules exhibiting loss of density or vacuolization was divided by the total number of granules. Degranulation indices were not evaluated by layer as the layers could not be precisely distinguished on EM.

### Clinical Outcome

A standardized history regarding the patient's symptoms was obtained pre-operatively and 30 days following cholecystectomy. The follow-up evaluation also included an assessment of the patient's global clinical response utilizing a Likert-type scale adapted to assess the change in pain by patient report. The five pain relief grades were:

Grade 1 Worse- clinical deterioration with increasing pain intensity and/or frequency.

Grade 2 No change- no increase or decrease in pain intensity or frequency

Grade 3 Moderate improvement- partial clinical response with definite improvement in pain, but not meeting the criteria for a Grade 4 response

Grade 4 Good- nearly complete relief of symptoms with minimal residual pain and pain not interfering with daily activities

Grade 5 Excellent- complete relief of pain

### Statistics

The frequencies for individual symptoms were compared between the pre- and post-cholecystectomy time points by chi square analysis or the Fisher's exact test. The percentage of patients with elevated peak and mean cell densities, respectively, was determined. The upper limit of normal was determined as the mean + 2 SD from previous control data with upper limits defined as mean > 3.5/hpf in the LP, peak > 5.78/hpf in the LP, mean > 1.66/hpf in the MM, and peak > 4.19 in the MM [7]. MC densities were compared between BD and CL patients by the student's t test. MC densities were compared between patients with and without chronic cholecystitis for all patients and for BD patients only by the student's t test. Pearson correlation coefficients were determined for the gallbladder ejection fractions with the MC densities and MC degranulation indices, respectively. Pearson correlation coefficients were determined for length of symptoms with the MC densities and MC degranulation indices, respectively. Mucosal MC densities were compared by layer between patients with a complete clinical response (grade 5) and lesser responders by ANOVA. A p value < .05 was considered significant.

## Results

Fifteen patients with BD and 13 symptomatic patients with CL who were scheduled for cholecystectomy were prospectively enrolled in this study. BD patients ranged in age from 8 to 17 years (mean 13.3 ± 2.7 years). Most were female (87%) and Caucasian (93%). CL patients ranged in age from 6 to 18 years (mean 12.2 ± 3.0 years). Again, most (85%) were female, 77% were Caucasian, 15% were African-American, and 8% were Hispanic. The presenting symptoms are shown in Table 1. For patients with BD, 66.7% had pain only in the right upper quadrant (RUQ), 6.7% only in the epigastrium, and 6.7% in both the RUQ and epigastrium. For CL patients, 69.2% had pain only in the RUQ, 15.4% only in the epigastrium, and 7.7% in both the RUQ and epigastrium. The remaining patients also had pain in other areas of the abdomen.

**Table 1:** The percentage of patients exhibiting particular symptoms preoperatively and 30 days post-cholecystectomy

	Cholelithiasis			Biliary Dyskinesia		
**Symptom**	**Pre**	**Post**	**p value**	**Pre**	**Post**	**p value**

Pain	100%	15.4%	< .01	100%	20%	< .01
Pain with eating	61.5%	15.4%	< .01	80%	6.7%	.04
Night waking with pain	84.6%	7.7%	.02	60%	6.7%	< .01
Nausea	100%	15.4%	< .01	80%	20%	< .01
Vomiting	53.8%	7.7%	< .01	40%	6.7%	.03
Diarrhea	23.1%	7.7%	.17	33.3%	6.7%	.17
Constipation	30.8%	46.2%	.40	20%	20%	1.00
Pain decreased with stool	7.7%	7.7%	1.00	26.7%	20%	1.00

**Figure 1:**
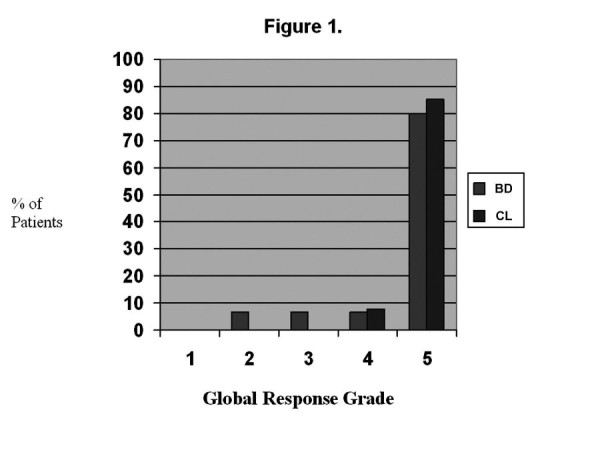
**Global clinical response grades for biliary dyskinesia (BD) and cholelithiasis (CL) patients 30 days post-cholecystectomy**.

The percentage of patients exhibiting a particular symptom at 30 days post-cholecystectomy is also shown in Table 1. One CL patient was lost to follow-up. There was a significant decrease in the frequency of pain, pain with eating, night time waking with pain, and nausea, respectively, in both patient groups. There was a significant decrease in vomiting frequency in the CL patients only. The global response grades are shown in Figure 1. Ninety- two percent of CL patients achieved a grade 4 or 5 response. Ninety-three percent of BD patients had a positive clinical response with 87% achieving a grade 4 or 5 response.

### Histology and Mast Cell Density

**Figure 2:**
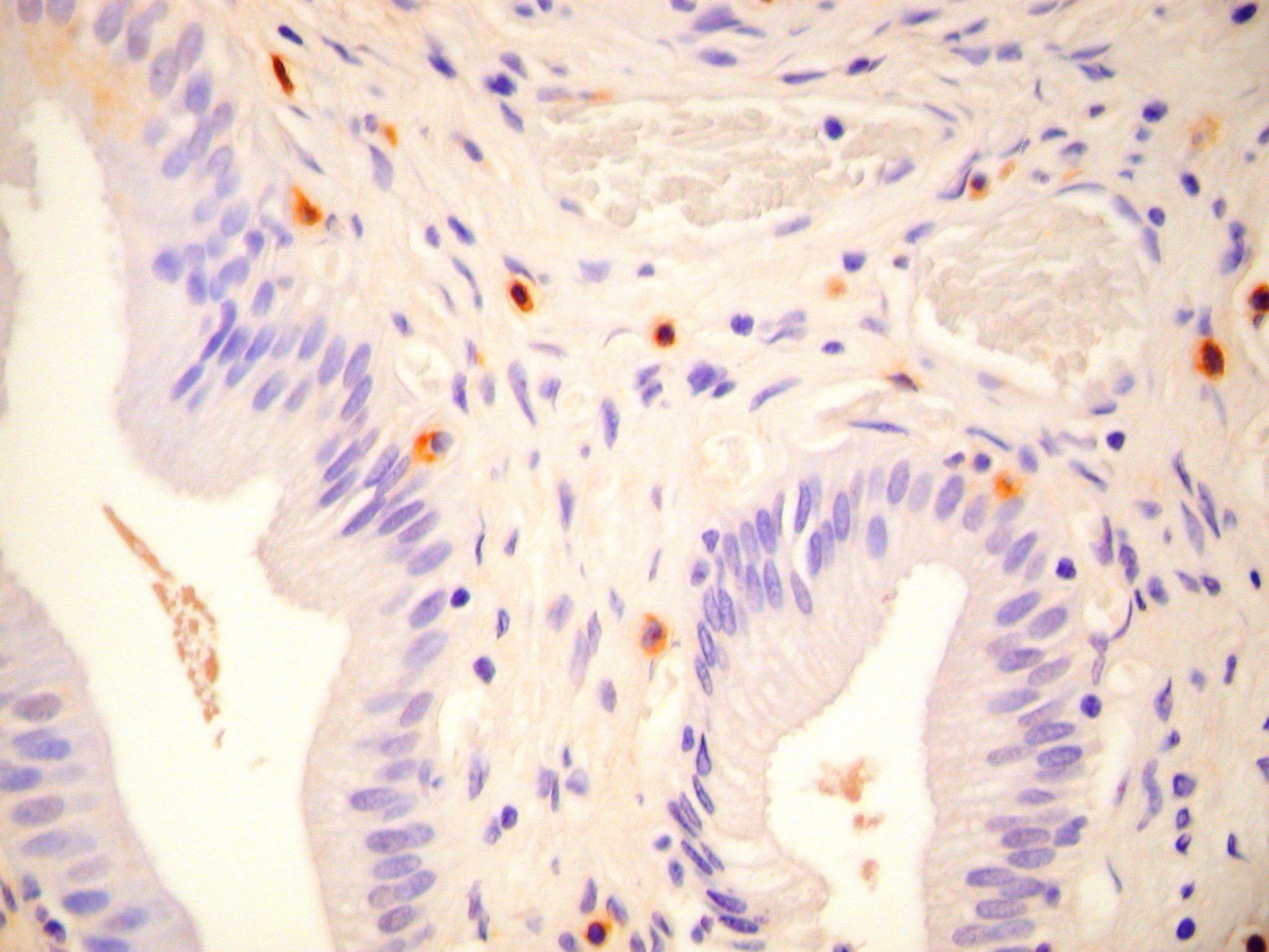
**Representative anti-tryptase stain in a patient with biliary dyskinesia**. (400X).

**Figure 3:**
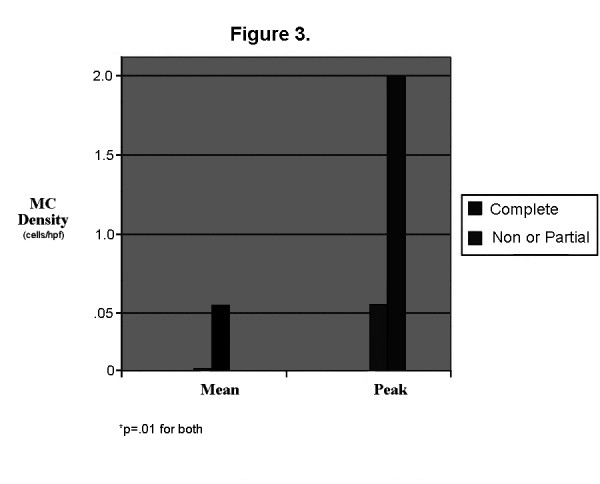
**Mean and peak epithelial mast cell densities in BD patients with a complete clinical response to cholecystectomy as compared to those with a partial response or non-responders**.

Chronic cholecystitis as determined by the pathologist was present in 85% of BD patients and 53% of CL patients. Pigmented stones were identified in 46.2% and cholesterol stones in 53.8% of the CL patients. The MC densities by layer are shown for both patient groups in Table 2. A representative IHC anti-tryptase stained specimen is shown in Figure 2. For CL patients, the means and peaks were elevated in all patients in both the LP and the MM. For BD patients, means were elevated in all patients in both the LP and MM and peaks were elevated in all patients in the LP and all but one patient in the MM. There was no significant correlation between the MC density and the ejection fraction. Both mean and peak epithelial MC densities were lower in complete responders (grade 5, N = 12) as compared to lesser responders (grades 2-4, N = 3) in BD patients. (Figure 3) There was no overlap in values between the 2 groups. The mean values in lesser responders ranged from 0.4 to 1.2/hpf while all complete responders had values ranging from 0 to 0.3/hpf. The peak values in lesser responders ranged from 2 to 4/hpf while complete responders had values ranging from 0 to 1/hpf. There were no differences in mast cell density in either the LP or the MM. There was no difference in density between pigmented and cholesterol stone patients. There were no differences in densities between patients with and without chronic cholecystitis for all patients combined or for BD patients only. For CL patients only, muscularis mucosae mean (r = 0.700, p = .017) and peak (r = 0.724, p = .012) MC densities correlated with length of symptoms.

**Table 2:** Mast cell densities by layer for cholelithiasis and biliary dyskinesia patients

Layer		Cholelithiasis	Biliary Dyskinesia	p value
Epithelium	mean	.36 ± .33	.25 ± .37	.43
	peak	1.18 ± .75	1.07 ± 1.20	.79
Lamina propria	mean	17.01 ± 7.92	11.90 ± 2.90	.035
	peak	24.55 ± 9.49	18.57 ± 4.69	.05
Muscularis	mean	11.33 ± 6.76	9.64 ± 3.94	.33
Mucosae	peak	17.91 ± 10.52	14.71 ± 5.25	.68

### Mast Cell Degranulation

**Figure 4:**
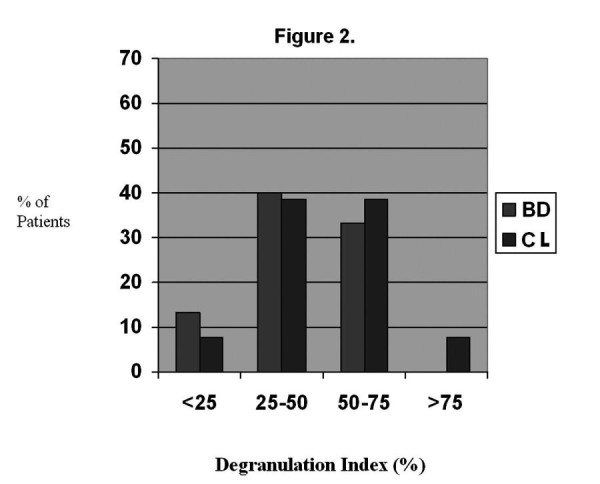
**Distribution of mast cell degranulation indices for biliary dyskinesia (BD) and cholelithiasis (CL) patients**.

**Figure 5:**
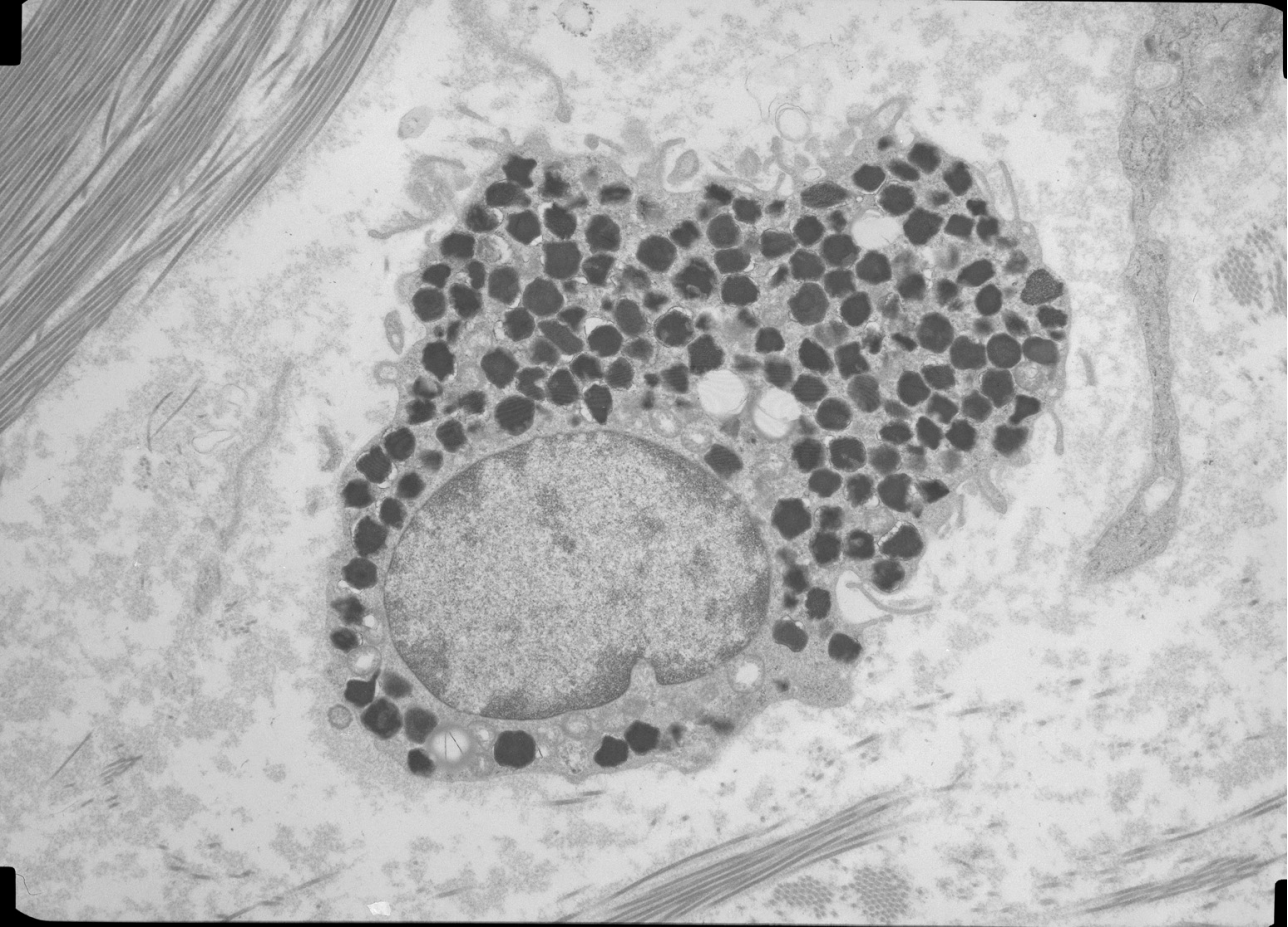
**Electron micrograph of a normal intact mast cell with homogenous electron-dense granules**.

**Figure 6:**
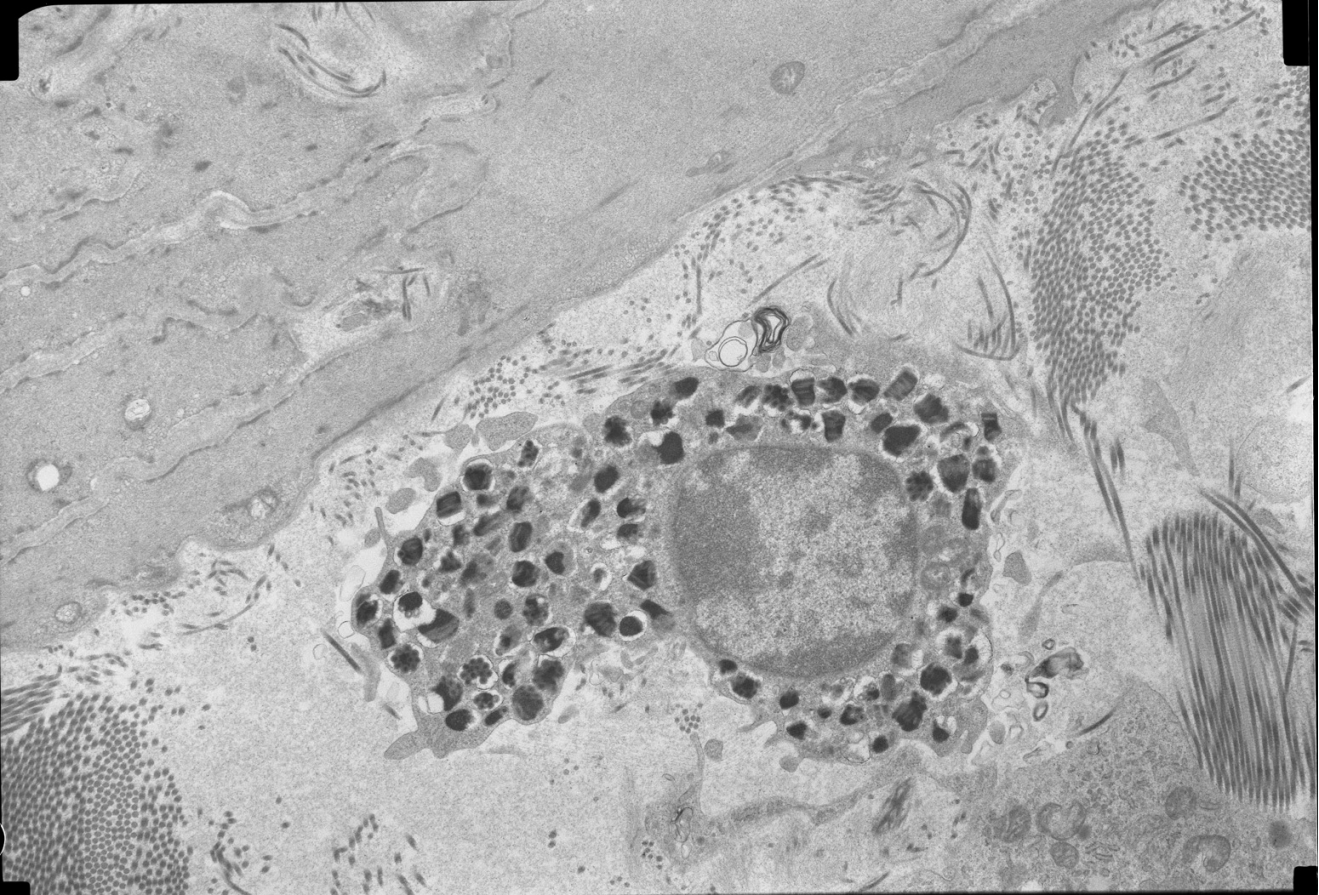
**Electron micrographs of mast cells demonstrating varying degrees of piecemeal degranulation evidenced by partially and completely empty granule chambers**.

**Figure 7:**
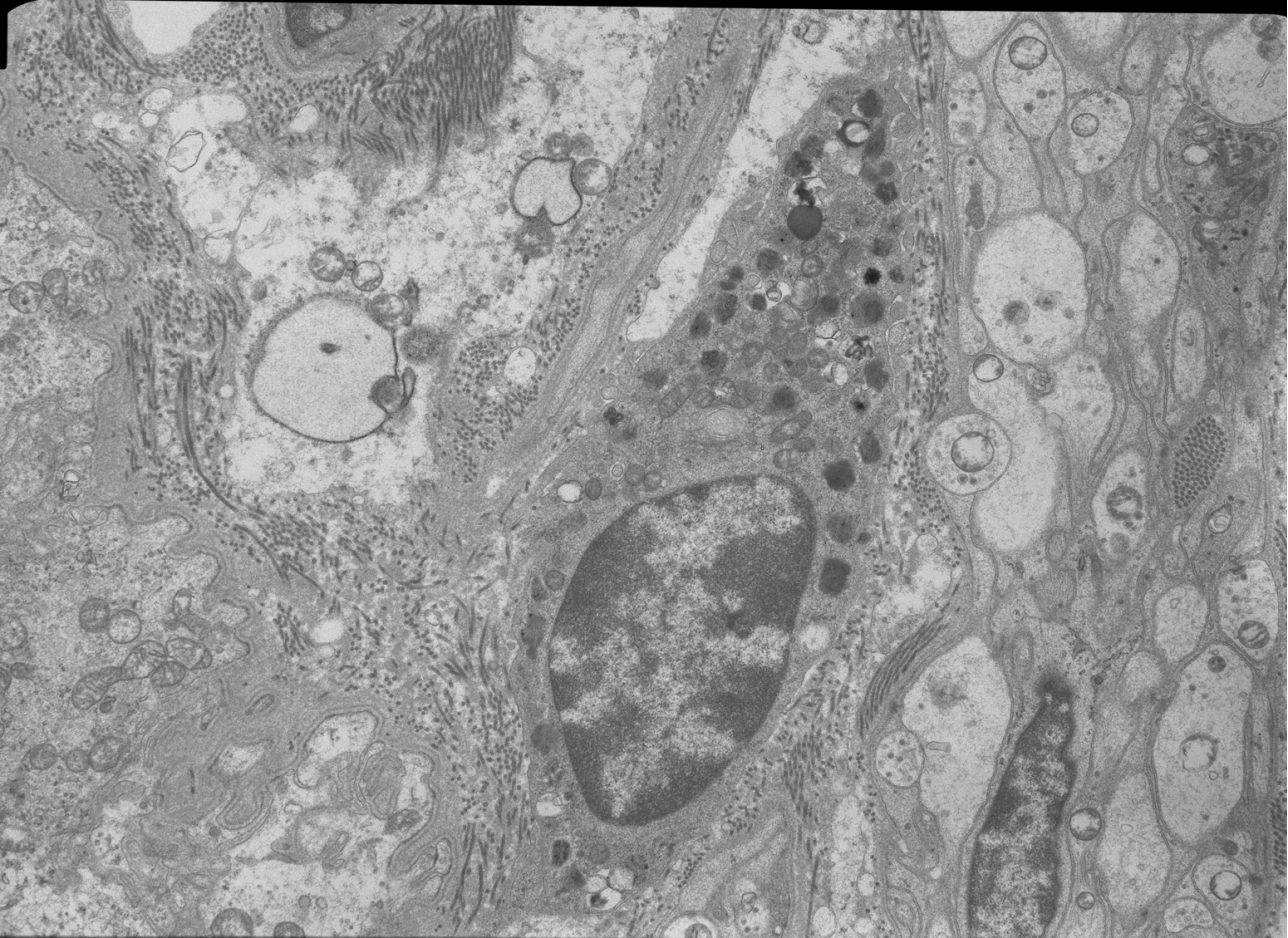
**Electron micrographs of mast cells demonstrating varying degrees of piecemeal degranulation evidenced by partially and completely empty granule chambers**.

**Figure 8:**
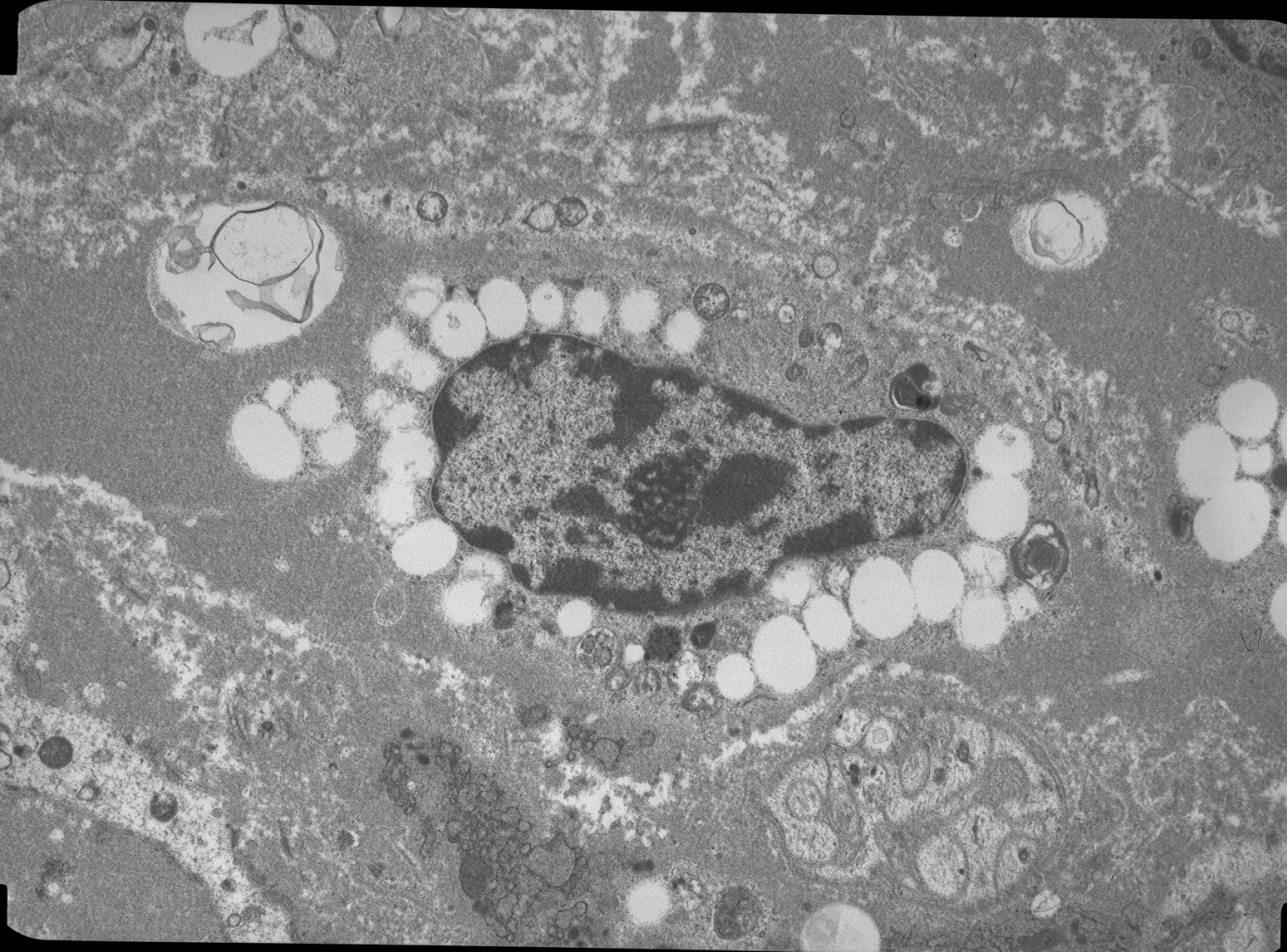
**Electron micrograph of a mast cell demonstrating nearly complete degranulation**.

A mean of 5.8 cells per patient (range 1-22) were evaluated. All but two patients had 3 or more cells evaluated. A mean of 458 granules per patient (range 71-1814) were evaluated. There was no correlation between the number of granules evaluated and the degranulation index (r = .000, p = .999). The overall MC degranulation indices were 49.4 ± 18.7% for the CL patients and 44.2 ± 16.8% for the BD patients. The comparative distribution of indices is shown in Figure 4. Overall, degranulation indices between 25 and 75% were seen in 77% of CL patients and 73% of BD patients. Representative electron micrographs of MCs are shown in Figures 5, 6, 7, and 8. Most MCs demonstrated piecemeal degranulation with variable numbers of partially and completely empty granules [15].

There was no significant correlation between MC degranulation and the ejection fraction. In BD patients only, there was a significant correlation between degranulation indices and both the mean (r = .534, p < .05) and peak (r = .632, p = .015) MC densities in the lamina propria. There were no differences between complete responders and lesser responders. Degranulation indices did not correlate with the length of symptoms in either CL or BD patients.

## Discussion

We have previously found that BD and symptomatic CL are associated with a 9- to 12-fold increase in gallbladder wall MCs when compared to controls [7]. In the current study, we confirmed that MCs are frequently present in the gallbladder wall at counts very similar to what was observed in our previous study. MCs have been implicated in other functional gastrointestinal disorders. Increased gastric MCs have been reported in adults with functional dyspepsia [8]. Increased MCs have been found in the ileum and colon of adults with irritable bowel syndrome [9-11]. Similar to our previous report, MC density was increased in the lamina propria and the muscularis mucosae in both CL and BD patients. For CL patients, this is similar to findings previously reported in adults with CL which demonstrated increased MC density and % of degranulated MCs in the muscular layer of adults with gallstones as compared to controls [16]. Unlike our previous report, MCs in the lamina propria were significantly increased in patients with CL as compared to patients with BD. The significance of this finding is not known. Moreover, it is possible that this represents a reaction to the stone itself or to an abnormal bile acid pool which may be present in stone formers. As we only studied symptomatic CL patients, it is not known whether MCs are unique to patients experiencing pain or whether they are also found in asymptomatic patients.

While we diagnosed BD in the current study utilizing adult criteria for functional gallbladder disorder, it should be noted that 85% of the patients in this group demonstrated chronic cholecystitis on pathologic examination. Our findings are consistent with other reports of pathology in biliary dyskinesia in children where chronic cholecystitis has been reported in 44-93% of patients [6,17,18]. This raises the question of whether these patients should be diagnosed with acalculous cholecystitis or whether chronic inflammation contributes to the pathogenesis or functional gallbladder disorders. This would be analogous to the increase in colonic lymphocytes reported in post-infectious irritable bowel syndrome [19]. Whether chronic inflammation is important in symptom generation is also unclear. In two previous studies, the proportion of patients with chronic cholecystitis did not differ between patients experiencing positive benefit from cholecystectomy as compared to those who did not [17,18].

A major focus of the current study was to define the degree of activation of gallbladder wall MCs by electron microscopy. The primary biologic and pathophysiologic actions of MCs result from the release of specific mediators which are often active in a concentration-dependent fashion. Thus the activity of MCs is defined by the density and degree of activation and degranulation [12]. Although there are no established norms for intestinal mast cell degranulation indices in the gastrointestinal tract, we have demonstrated a moderate to high degree of activation comparable to that seen in other diseases where MCs are known to have a pathophysiologic role [14]. MC products have the potential to result in pain through direct stimulation of sensory nerves or induction of visceral hyperalgesia. MC activation has been associated with pain severity and increased visceral sensitivity in adults with both functional dyspepsia and irritable bowel syndrome [11,14,20]. MC degranulation results in a decreased threshold for the pain response with balloon distension and this hyperalgesic response can be prevented with MC-stabilizing medication [21] MC may also create gallbladder symptoms through the generation of gallbladder spasm. Two MC products, histamine and leukotrienes, have been shown to increase the excitability of gallbladder smooth muscle and lead to contraction [22-25]. We were unable to demonstrate a correlation between MC density or activation and the ejection fraction although this may be the result of the sensitivity of the method employed. The activation index is a general indicator of activation but may be limited in assessing specific pathophysiologic processes. MCs produce many mediators which may be selectively released with varying physiologic actions. Additionally, while EM activation has been described in conditions where MCs appear to play a role, there is no "gold standard" measure of MC activation which would allow assessment of the accuracy of degranulation indices. Further work is necessary to determine if specific mediators are responsible for the observed dysmotility.

Another focus of the current study was to prospectively evaluate global response, to determine which symptoms improve with cholecystectomy, and to evaluate whether there is a relationship between MC density or activation and clinical response. Consistent with previous reports, a complete or nearly complete clinical response was seen in all CL patients. Specific symptoms decreasing in frequency were abdominal pain, pain with eating, night waking with pain, nausea, and vomiting while lower tract symptoms (diarrhea, constipation, and pain improving with a stool) did not improve. Cholecystectomy resulted in a complete clinical response in 80% of BD patients, with an additional 15% experiencing clinical improvement. Specific symptoms decreasing in frequency were abdominal pain, pain with eating, night waking with pain, and nausea. Again, lower tract symptoms did not decrease in frequency postoperatively. Our response rate for BD is consistent with previous studies, most of which have been retrospective. As reviewed by Constantinou, et al, eleven previous studies reported postoperative response rates varying from 71-100% [17]. Constantinou, et al, also reported outcomes following cholecystectomy in 100 children with BD where outcome data were collected prospectively [17]. Similar to our patients, 77% reported resolution of symptoms at 6 months to 5 years postoperatively. The only randomized trial for BD was performed in adults [26] This study found cholecystectomy to be more efficacious than observation. Siddiqui, et al, retrospectively evaluated the degree of response to cholecystectomy for BD in children [27]. Similar to our patients, the overall response rate was 93% with 65% reporting resolution of symptoms and 28% reporting improvement with some residual symptoms. Overall, our study adds to the previous prospective study and confirms the clinical efficacy that has been reported in the previously cited retrospective studies.

The only relationship between MC activation or density and the clinical response grade for either patient group was demonstrated in the BD patients. A complete clinical response was associated with lower epithelial MC density when compared to a partial or non-response. The reason for this is not clear. It is possible that higher MC density may be associated with MCs in the gastrointestinal tract resulting in dyspepsia or irritable bowel syndrome. This could not be evaluated in the current study as only a few patients underwent endoscopic evaluation. There are insufficient data to determine whether there is clinical value to obtaining gastrointestinal biopsies prior to cholecystectomy. These data (both the positive findings in the epithelium and the negative findings in other layers) need to be interpreted with caution as there were so few partial or non-responders for comparison. The positive findings may simply represent a type II statistical error. Larger studies are necessary to evaluate the relationship between MCs and outcome.

## Conclusions

In the current study, we have demonstrated a moderate to high degree of mast cell activation in BD and CL which along with the previously demonstrated high MC density (confirmed in the current study) suggests a possible role for MCs in the pathophysiology of these conditions. Future work evaluating concentrations of gallbladder wall mediators such as proteases or cytokines is necessary to further delineate possible pathophysiologic mechanisms. We have demonstrated a relationship between MC density and outcome for biliary dyskinesia which also warrants further investigation. A better understanding of the pathophysiology may lead to better identification of patients who will have clinical benefit from cholecystectomy or who might respond to medications such as mast cell stabilizers.

## List of Abbreviations

MC: mast cell; BD: biliary dyskinesia; CL: cholelithiasis; FGD: functional gallbladder disorder; IBS: irritable bowel syndrome; RUQ: right upper quadrant; CCK: cholecystokinin; IHC: immunohistochemical; EM: electron microscopic; LP: lamina propria; MM: muscularis mucosae.

## Competing interests

The authors declare that they have no competing interests.

## Authors' contributions

CF conceived of the study, analyzed the data, and wrote the first draft of the manuscript. All authors participated in the design of the study and critically revised the manuscript. NN performed all of the lab work. Authors read and approved the final manuscript.
